# Medical College of Ohio

**Published:** 1835

**Authors:** 


### II. Medical College of Ohio—New Board of Trustees.

The General Assembly of Ohio have at length opened their eyes to the "low estate" of their Medical College, and appointed a new Board of Trustees, consisting of the following gentlemen:

William H. Harrison
Morgan Neville
John C. Wright
William S. Hatch
William Burke,
William Stevenson
Calvin Fletcher
A. H. Dunlavy, of Lebanon
Dr. Laomi Rigdon, of Hamilton
" Joseph S. Carter, of Urbana
" Samuel Parsons, of Columbus
" John Cotton, of Marietta
" Alexander Campbell, of Ripley.

